# The Toxicity of Tire Wear Particles and Their Leachates on Digestion and Gut Microbiota of *Mytilus coruscus*

**DOI:** 10.3390/toxics14060468

**Published:** 2026-05-27

**Authors:** Yu Zhou, Qikun Yang, Lukuo Ma, Xuanjie Zhou, Shixiu Wang, Wei Huang

**Affiliations:** 1 Ocean College, Zhejiang University, Zhoushan 316021, China; 18845869800@163.com (Y.Z.); 12434070@zju.edu.cn (Q.Y.); 2 Key Laboratory of Ocean Space Resource Management Technology, Ministry of Natural Resources, Hangzhou 310012, China; xvh593591@163.com (L.M.); 3200100401@zju.edu.cn (X.Z.); 3 Key Laboratory of Marine Ecosystem Dynamics, Second Institute of Oceanography, Ministry of Natural Resources, Hangzhou 310012, China; shixiuwang3@163.com; 4 State Key Laboratory of Satellite Ocean Environment Dynamics, Second Institute of Oceanography, Ministry of Natural Resources, Hangzhou 310012, China; 5 Key Laboratory of Nearshore Engineering Environment and Ecological Security of Zhejiang Province, Second Institute of Oceanography, Ministry of Natural Resources, Hangzhou 310012, China; 6 National Demonstration Center for Experimental Fisheries Science Education, Shanghai Ocean University, Shanghai 201308, China

**Keywords:** mussel, tire wear particle, leachate, gut microbiota, Integrated Biomarker Response

## Abstract

Tire Wear Particles (TWPs) are a major type of microplastics (MPs). However, previous studies have predominantly focused on TWP leachates rather than the particles, and their toxic effects on marine organisms remain limited. In this study, the mussels were exposed to TWPs and their leachates for 21 days, followed by a 7-day recovery period in clean conditions. The results showed that the leachates contained organic pollutants (predominantly PAHs) and metal ions (predominantly Zn^2+^). Growth inhibition was observed in exposure to TWPs, while leachate exposure showed no significant effect. As for the antioxidant system, high-concentration TWP and their leachate exposures provoked significant oxidative stress, accompanied by inhibited superoxide dismutase (SOD) and catalase (CAT) activity as well as increased glutathione (GSH) and malondialdehyde (MDA) levels. Exposure to high concentrations of TWPs significantly inhibited acetylcholinesterase (AChE) and trypsin (TRS) activities. Gut microbiota analysis indicated that exposure to TWPs and their leachates modified community structure with significantly reduced relative abundance of Firmicutes, Bacteroidota, potentially attributed to the bacteriostatic activity of released Zn^2+^. Integrated Biomarker Response (IBR) analysis indicated that TWPs elicited stronger integrated toxicity compared to their leachates. This study provides a comparative perspective on the ecological toxicology of TWPs and their leachates.

## 1. Introduction

Microplastics (MPs, diameter ≤ 5 mm) are widely distributed in the environment and have become a global pollution issue [1]. Recent studies indicate that tire wear particles (TWPs) may constitute a primary contributor to marine microplastic pollution [2,3,4,5], with concentrations in surface waters reaching 6.3 mg/L [6] and in sediments ranging from 0.3 to 155 g/kg, accounting for as high as 60% of total microplastic contamination in surface waters and sediments [7,8,9,10]. Tire wear has been amplified by rising vehicle populations and associated increases in tire production. During the interaction between tires and the road surface under mechanical abrasion, a mixture of particles ranging in size from nanometers to several hundred micrometers is generated, referred to as TWP. It is estimated that the average annual emission of TWP was 0.81 kg per capita, with an annual global emission amounting to approximately 6 million tons [3]. Notably, TWPs have garnered significant attention due to their widespread distribution and ecological risks [11], and they exhibit reduced degradability due to the formation of three-dimensional cross-linked structures during the vulcanization process [12]. TWPs are transported via atmospheric pathways and subsequently enter aquatic systems through runoff [4], leading to accumulation and potential threats to aquatic organisms. However, current studies of their toxic effects remain limited; most studies have focused on acute exposure to tire leachates [13] on organisms such as copepods [14], daphnids [15], algae [7], and fishes [16].

Once entered into ocean ecosystems, TWP can be ingested by marine organisms [17,18], with detrimental consequences for their health and survival. At the same time, chemical substances in the leachates are also harmful to marine organisms [17,19]. Compounds identified in TWP leachates include heavy metals (e.g., zinc, lead, cadmium) and organic pollutants such as polycyclic aromatic hydrocarbons and benzothiazole derivatives, which are primarily introduced during the manufacturing process [20,21]. Furthermore, TWPs can release adsorbed pollutants into water environments [14]. TWPs pose ecological risks to marine organisms through both physical ingestion and chemical exposure. Their leachates can disrupt behavior [22], induce oxidative stress [23], affect reproductive development [24], disrupt the immune system [23], interfere with the endocrine system [22], impair digestion [14], and change gut microbiota [25]. Consequently, the potential ecological risks associated with TWPs cannot be overlooked.

Bivalves could readily accumulate pollutants through their filtration activities, and their high sensitivity to pollutants supports their value as sentinel species for assessing marine environmental health [26]. Their substantial filtration capacity allows populations to process the entire water column multiple times daily, accumulating pollutants in the surrounding environment, including MPs and heavy metals. This process fundamentally shapes nutrient cycling and enables benthic-pelagic coupling in coastal ecosystems [27]. However, this efficient particle-capturing mechanism also renders them highly vulnerable to microplastic pollution. Over the past few years, extensive research has been conducted on the effects of TWP leachates on bivalves. However, the impact of particles remains poorly understood. Given the ecological relevance of bivalves in coastal ecosystems, more research is needed to assess the toxicity of TWP pollution to this key species.

To compare the toxicity of TWPs and their leachates, we performed experiments with multiple concentration levels of particles and leachates. We investigated underlying mechanisms by measuring oxidative stress, neurotoxicity, and digestive enzyme biomarkers. The integrated toxicity was synthesized using the IBR index to quantify and contrast the overall toxic pressure. Furthermore, we analyzed changes in the gut microbiota to elucidate the potential toxicity mechanism.

## 2. Materials and Methods

### 2.1. Experimental Organisms

Mussels (73.86 ± 5.74 mm shell length, 34.13 ± 56.24 g wet weight) used in the experiment were collected from Gouqi Island (Zhejiang, China, 30°42′ N, 122°46′ E). Prior to the experiment, the mussels were acclimated in the experimental aquaculture system for 14 days under controlled conditions: temperature of 25 ± 0.4 °C, salinity of 28 ± 0.8, and a 12:12 h light-dark cycle. During acclimation, the microalgae *Chlorella vulgaris* were supplied daily at a concentration of 5 × 10^4^ cells/mL. The filtered seawater was renewed every day, and dead mussels were discarded.

### 2.2. Preparation of TWPs and Their Leachates

Recycled and crushed waste truck tire particles were purchased from Huayi Rubber Co., Ltd. (Dujiangyan, China). TWPs were first sieved through a 100 μm mesh, and the sieved particles were collected for experimental use. These particles were then shaken in artificial seawater for 14 days, rinsed with ultrapure water, and selected to obtain the final experimental particles. One hundred TWPs were randomly selected, and their average particle size was determined using ZEN Lite software (Carl Zeiss Microimaging GmbH, Oberkochen, Germany, version 6.0.6874). The average particle diameter was determined to be 60.86 ± 43.97 μm, with the fraction of 0–30 μm constituting the largest proportion (Figure S1). The surface morphology of TWPs was characterized by confocal microscopy (SteREO Discovery V8, Carl Zeiss, Oberkochen, Germany) and scanning electron microscopy (SEM; Hitachi JSM-7500F, Hitachi High-Technologies Corporation, Hitachi, Japan). We distinguished the TWPs in this study from other microplastics based on their unique morphology. TWPs presented irregular, angular shapes and rough, heterogeneous textures, while conventional plastic fragments tended to exhibit smoother, more rounded, or regularly fractured forms.

The TWP leachates were prepared following the method of Yang et al. (2022) [14]. Treated TWPs (post shaking, rinsing, and sieving) were added to artificial seawater (salinity = 28 ± 0.1) filtered through a glass-fiber membrane (GF/F; pore size: 0.7 μm; diameter: 47 mm) to prepare stock leachates with a concentration of 10 g/L. The mixture was poured into 1.5 L glass conical flasks, ultrasonicated for homogenization, and subsequently shaken in a constant temperature incubator shaker (HZQ-120H, Shanghai Yiheng Scientific Instrument Co., Ltd. Shanghai, China) at 25 °C and 200 rpm for 14 days. After shaking, the suspension was filtered through a glass-fiber membrane, and the resulting filtrate was collected and stored at 4 °C as the stock leachates. The filtered leachates were then analyzed for metal concentrations using inductively coupled plasma mass spectrometry, and for organic compounds using gas chromatography-mass spectrometry (see Text S1 for details).

### 2.3. Experimental Design

To investigate the toxic effects of TWPs and their leachates, four concentration levels were established for each exposure type: TWP suspensions (0, 1, 10, and 100 mg/L) and TWP leachates (0%, 0.01%, 0.1%, and 1%, diluted from the stock leachates). After acclimation, mussels were randomly divided into 8 groups, including 4 TWP and 4 leachate groups, with each treatment comprising three replicates (30 L aquariums, *n* = 30 mussels per aquarium). The exposure experiment spanned 21 days, followed by a 7-day recovery period specifically for the intestinal microbial analysis group. Mussel samples were collected at four time points (0, 7, 14, and 21 days) to assess time-dependent biological responses. At each sampling time point, three mussels per aquarium were pooled to form one biological replicate (*n* = 3) for biochemical assays. For gut microbiota analysis, four mussels per aquarium were pooled to form one biological replicate (*n* = 3). After collection, all samples were immediately frozen in liquid nitrogen and stored at −80 °C until analysis. Both TWPs and their leachates were administered with the same concentration gradient to allow for a comparative assessment of their toxic effects. The stored stock leachates were used for daily renewal to ensure the maintenance of required concentrations. The culture medium in each aquarium was completely renewed daily with fresh working solutions of the target concentrations. Additionally, mussel growth rates were measured under the exposure conditions, with gills collected for subsequent analyses of other indicators.

### 2.4. Growth Rate

Mussel shell length was measured on days 0 and 21, and the specific growth rate (SGR) was calculated using the following formula:

(1) $$SGR=\frac{lnL_{2}-lnL_{1}}{t}\times 100\%$$

where *L*_2_ represents the shell length on day 21, *L*_1_ represents the shell length on day 0, and *t* represents the number of days between the two measurements of *L*_2_ and *L*_1_.

### 2.5. Biochemical Indicator

At each sampling time point, three mussels were randomly selected from each aquarium for subsequent enzyme activity analysis. To minimize individual variability, the three samples from the same tank were pooled as a single biological replicate, placed in cryotubes, and immediately frozen in liquid nitrogen. The gill samples were homogenized in an ice-water bath with nine volumes of normal saline (*w*/*v* = 1:9). The homogenates were centrifuged at 2500 rpm (~770× *g*) for 10 min, and the supernatants were collected for biochemical analysis. Biochemical parameters were quantified using commercial assay kits (Nanjing Jiancheng Biotechnology Research Institute, Nanjing, China). The assay principles are briefly described as follows. Superoxide dismutase (SOD) activity was determined by the inhibition of the photochemical reduction of nitroblue tetrazolium (NBT) at 560 nm. Catalase (CAT) activity was measured by monitoring the decomposition of H_2_O_2_ at 240 nm. Reduced glutathione (GSH) content was assessed using 5,5’-dithiobis-(2-nitrobenzoic acid) (DTNB), which reacts with GSH to produce a yellow compound measured at 412 nm. Malondialdehyde (MDA) level, an indicator of lipid peroxidation, was determined by its reaction with thiobarbituric acid (TBA) to form a pink chromogen measured at 532 nm. α-Amylase (AMS) and trypsin (TRS) activities were measured using starch and casein as substrates, respectively, with the release of reducing sugars or an increase in absorbance at specific wavelengths. Acetylcholinesterase (AChE) activity was assayed based on the hydrolysis of acetylcholine to thiocholine, which reacts with DTNB to produce 5-thio-2-nitrobenzoate measured at 412 nm. The BCA method normalizes all enzyme activity and biomarker content data, which are expressed as units per milligram of protein (hereafter ‘prot’ refers to protein). The assays were validated using standard curves provided with the kits (correlation coefficient R^2^ > 0.99 for all assays). Absorbance was measured using a microplate reader (Thermo Scientific Varioskan LUX, Waltham, MA, USA), and the concentrations of the respective biochemical indicators were calculated according to the manufacturers’ protocols.

### 2.6. Gut Microbiota

At each sampling time point, four mussels were randomly selected from each aquarium for intestinal microbiome analysis. To minimize individual variability, the four samples from the same tank were pooled as a single biological replicate, placed in a cryotube, frozen in liquid nitrogen, and stored at −80 °C. Total microbial community DNA was extracted from the sample using a QIAamp DNA mini kit (QIAGEN, Düsseldorf, Germany). DNA quality and purity were assessed by 1% agarose gel electrophoresis and a NanoDrop 2000 spectrophotometer (Thermo Fisher Scientific, Waltham, MA, USA), respectively. The V3–V4 hypervariable region of the bacterial 16S rRNA gene was amplified with the primer pair 338F/806R. The PCR products were purified by 2% agarose gel electrophoresis, recovered, and quantified using a Qubit 4.0 Fluorometer (Thermo Fisher Scientific, Waltham, MA, USA). Sequencing was performed on the Illumina NextSeq2000 (Illumina, Inc., San Diego, CA, USA) platform. Raw reads were quality-filtered using fastp. Paired-end reads were merged using FLASH (minimum overlap of 10 bp, maximum mismatch rate of 0.2, version 1.2.11) and assigned to samples based on their unique barcodes and primers. UPARSE (Robert C. Edgar, CA, USA) was used to cluster sequences into operational taxonomic units (OTUs) at a 97% similarity threshold, and chimeric sequences were removed. To standardize sequencing depth, sequences from each sample were rarefied to 20,000 reads, resulting in a Good’s coverage of 99.09%. Taxonomic annotation was performed using the RDP classifier with the SILVA database (v138) at a 70% confidence threshold. Functional profiles of the microbial communities were predicted using PICRUSt2 (version 2.5.2).

### 2.7. Integrated Biomarker Response (IBR)

To assess the effects of TWPs and leachate exposure on enzyme activities, the parameters SOD, CAT, GSH, MDA, AMS, TRS, and AChE were integrated into the IBR index on day 21 of the experiment. According to the research findings of Devin et al. (2014) [28], the calculation of the IBR value involves three steps:

(2) $$P=\frac{z-m}{s}$$

where *P* represents the standardized value of the biomarker, *z* represents the response value of each biomarker, *m* represents the average value of the biomarkers, and *s* represents the standard deviation of the biomarkers.

(3) Y=P+|min|

where *P* is in the activated state, it exerts a +*P* effect; otherwise, it exerts a −*P* effect in the inhibited state. |*min*| represents the absolute minimum *P* value for each biological marker.

The *Yi* values were plotted as a star graph, and the *IBR* was calculated as the sum of the areas of the triangles defined by the *Y* standardized biomarkers.

(4) $$IBR=\sum_{i=1}^{k}\frac{Y_{i}\times Y_{i+1}\times \sin\left(\frac{2\pi }{k}\right)}{2}$$

The IBR index was calculated, and a star-shaped diagram was constructed for each treatment group based on the response values of the 7 biomarkers.

### 2.8. Statistical Analysis

All data are expressed as the mean ± standard deviation (n = 3). The normality of the data distribution was assessed via the Kolmogorov–Smirnov test in Origin Pro 2021. Two-way ANOVA was used to analyze the effect of time and TWP concentrations. Student’s t-test was used to compare the differences in physiological parameters between TWPs and their leachates under the same concentration, and one-way ANOVA was used to compare the effects of different concentrations of TWPs and their leachates at the same time points if an interaction was observed. When a significant overall effect was observed (*p* < 0.05), differences between individual treatment groups and the control were assessed using Tukey’s HSD test for post hoc multiple comparisons. Alpha diversity indices (Chao1, Shannon, Simpson) were analyzed with the QIIME 2 q2-diversity plugin, visualized via ggplot2 in R, and statistically compared using one-way ANOVA with Tukey’s post hoc test (*p* < 0.05). Beta diversity was assessed based on Bray–Curtis dissimilarities, ordinated by PCoA, and statistically validated by PERMANOVA in the R vegan package. LEfSe (LDA threshold = 2.0) was used to identify differentially abundant taxa at the phylum and genus levels. PICRUSt2 was applied for functional prediction of microbial communities to infer KEGG Level 2 pathways, with differential enrichment analyzed by the Kruskal–Wallis test and Benjamini–Hochberg correction. Additionally, Pearson’s correlation coefficient (r) was used to analyze the correlations between the variation in various biomarkers. For all statistical analyses, *p* < 0.05 was defined as statistically significant. All experimental results were visualized using Origin Pro 2021.

## 3. Results

### 3.1. TWPs and Their Leachate

The surface of the TWPs exhibited a rough and uneven morphology with distinct depressions and protrusions, a feature attributable to the tire wear process during vehicle operation and the subsequent particle preparation procedures. The stock solution of the leachates prepared from TWPs was identified to contain a variety of organic pollutants and heavy metal ions (Table S1). Among the polycyclic aromatic hydrocarbons (PAHs) detected, naphthalene and 2-methylnaphthalene were found to have the highest concentrations, respectively, at 38.89 μg/L and 31.00 μg/L. For heavy metal ions, Zn^2+^ exhibited the highest concentration, reaching 1289.99 μg/L.

### 3.2. Growth Inhibition of TWPs on Mytilus coruscus

The growth rates of mussels in different groups are presented in Figure S2. The results demonstrated that the growth rates of mussels were significantly lower (*p* < 0.05) in both the 10 mg/L and 100 mg/L TWP groups compared with the control (Figure S2A). However, there was no significant difference in mussel growth rates among all groups exposed to different concentrations of the TWP leachates (Figure S2B).

### 3.3. Effects of TWPs and Leachates on Physiological Indexes

#### 3.3.1. Effects of Particulate Exposure

**Figure 1:**
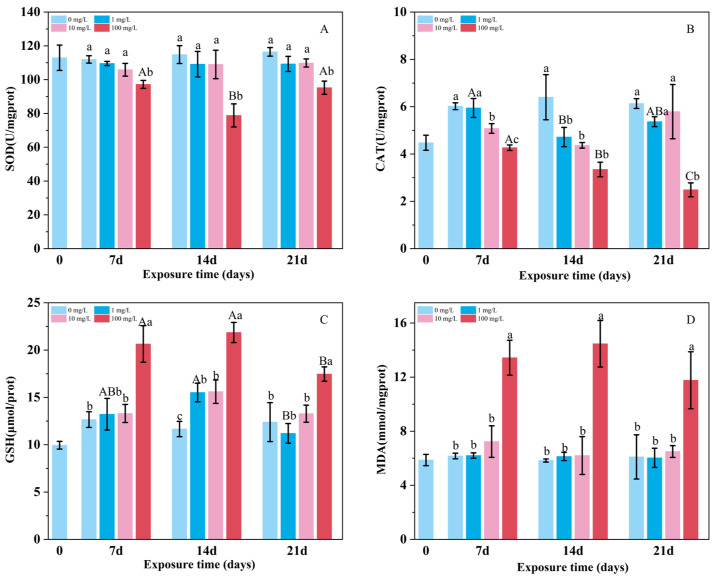
Influence of TWPs on (**A**) superoxide dismutase (SOD), (**B**) catalase (CAT), (**C**) glutathione (GSH), and (**D**) malondialdehyde (MDA) (different uppercase letters indicate significant differences at the same concentration across different time points between control groups and experimental groups; different lowercase letters indicate significant differences at the same time point across different concentrations between control groups and experimental groups).

SOD activity exhibited a decreasing trend with increasing concentration. On days 7, 14, and 21, SOD activity in the high-concentration (100 mg/L) group was significantly lower (*p* < 0.05) than that in the control group, low-concentration (1 mg/L) group, and medium-concentration (10 mg/L) group, with the activity reaching its minimum on day 14 (Figure 1A). CAT activity showed a similar variation pattern, and the high-concentration group exerted the most significant inhibitory effect on CAT activity. Temporal variations in CAT activity were also observed. In the low-concentration group, CAT activity on day 14 differed significantly from that on days 7 and 21 (*p* < 0.05); in the medium-concentration group, CAT activity on day 21 was significantly different from that on days 7 and 14 (*p* < 0.05) (Figure 1B). In contrast, GSH level increased with the rise in particle concentration, and the high-concentration group had significantly higher (*p* < 0.05) GSH level than other groups. In the low-concentration group, GSH level on day 21 was significantly higher (*p* < 0.05) than that at the earlier time points (Figure 1C). MDA content, a biomarker for lipid peroxidation, presented a variation pattern similar to GSH, with the highest levels detected in the high-concentration group, which were significantly higher (*p* < 0.05) than those in other groups (Figure 1D).

**Figure 2:**
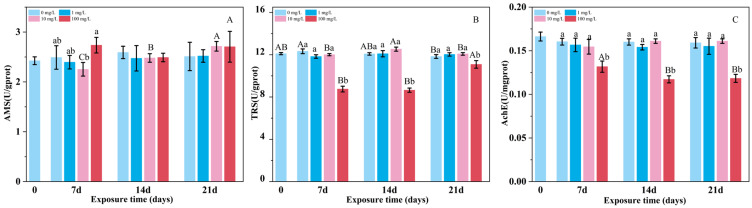
Influence of TWPs on (**A**) α-amylase (AMS), (**B**) trypsin (TRS), and (**C**) acetylcholinesterase (AChE) (different uppercase letters indicate significant differences at the same concentration across different time points between control groups and experimental groups; different lowercase letters indicate significant differences at the same time point across different concentrations between control groups and experimental groups).

AMS activity increased with the rise in particle concentration. On day 7, the medium-concentration treatment exhibited the lowest AMS activity, which was significantly different from that in the high-concentration groups (*p* < 0.05) (Figure 2A). In contrast, TRS activity decreased with the increase in exposure concentration, with the lowest values detected in the high-concentration group. Additionally, in the medium-concentration group, TRS activity on day 14 differed significantly from that on days 7 and 21 (*p* < 0.05) (Figure 2B). AChE activity also showed a concentration-dependent inhibitory trend, and the high-concentration group significantly inhibited AChE activity compared to other groups (*p* < 0.05). The lowest AChE activity among all groups was recorded on day 14 (Figure 2C).

#### 3.3.2. Effects of Leachate Exposure

**Figure 3:**
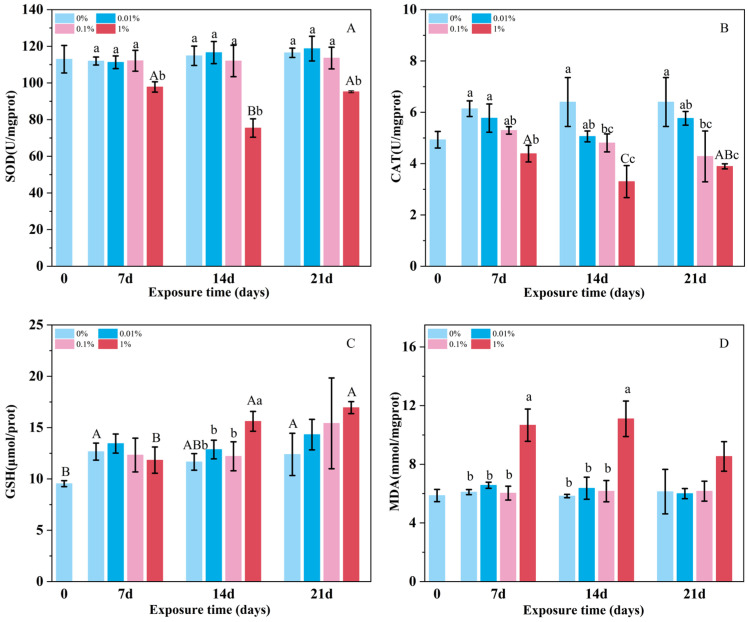
Influence of TWP leachates on (**A**) SOD, (**B**) CAT, (**C**) GSH, and (**D**) MDA (different uppercase letters indicate significant differences at the same concentration across different time points between control groups and experimental groups; different lowercase letters indicate significant differences at the same time point across different concentrations between control groups and experimental groups).

SOD activity showed a decreasing trend with increasing leachate concentration. The high-concentration (1%) treatment significantly suppressed SOD activity compared to the control, low-concentration (0.01%), and medium-concentration (0.1%) groups (*p* < 0.05), with the lowest value observed on day 14 (Figure 3A). A similar inhibitory pattern was observed for CAT activity, which was also lowest in the high-concentration group. CAT activity reached its minimum level on day 14 across all concentration groups (Figure 3B). In contrast, GSH level increased in a concentration-dependent manner. The high-concentration treatment induced significantly higher (*p* < 0.05) GSH level than all other groups (Figure 3C). MDA, indicative of lipid peroxidation, exhibited a trend similar to GSH. The high-concentration group resulted in significantly elevated MDA levels compared to other groups (*p* < 0.05), with the peak value recorded on day 14 (Figure 3D).

**Figure 4:**
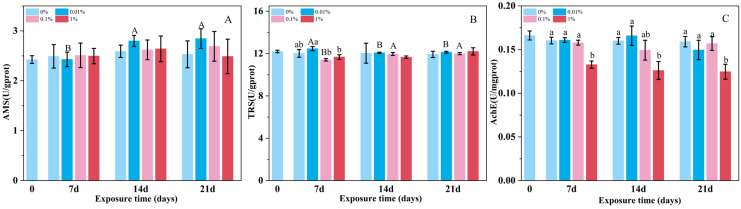
Influence of TWP leachates on (**A**) AMS, (**B**) TRS, and (**C**) AChE (different uppercase letters indicate significant differences at the same concentration across different time points between control groups and experimental groups; different lowercase letters indicate significant differences at the same time point across different concentrations between control groups and experimental groups).

AMS activity demonstrated a biphasic response to the increase in leachate concentration, characterized by an initial increase followed by a subsequent decrease. On day 7, the low-concentration group displayed the lowest AMS activity, which was significantly different from that in the medium-concentration and high-concentration groups (*p* < 0.05) (Figure 4A). TRS activity was significantly affected at a specific time point, on day 7, TRS activity in the medium and high-concentration groups were significantly different from those in the control group (*p* < 0.05) (Figure 4B). AChE activity exhibited a clear concentration-dependent inhibitory trend, with the high-concentration group significantly inhibiting AChE activity (Figure 4C).

#### 3.3.3. The IBR of Mussels to TWPs and Their Leachates Exposure

**Figure 5:**
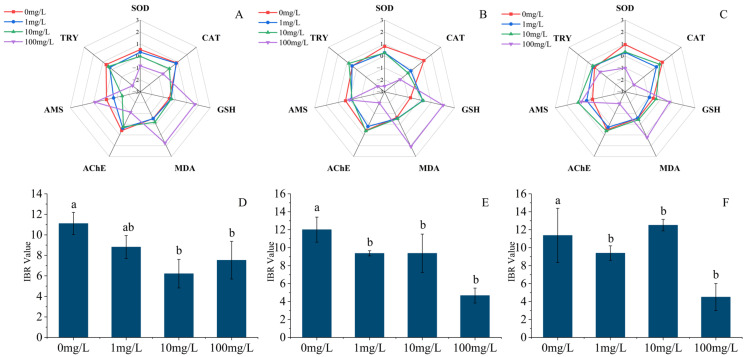
Star plots (**A**–**C**) and corresponding Integrated Biomarker Response (IBR) values (**D**–**F**) of physiological and biochemical indices in mussels exposed to TWPs for 7, 14, and 21 days ((**A**,**D**): 7 days; (**B**,**E**): 14 days; (**C**,**F**): 21 days, different letters mean there are significant differences between control groups and experimental groups).

Based on the results of the IBR index, significant differences were observed between the exposure groups and the control group on days 7, 14, and 21. Compared to the control group, the high-concentration TWP groups exerted a notable impact on the activities of enzymes associated with physiological oxidative stress, digestion, and neurotoxicity in mussels (Figure 5D–F). In contrast, the low-concentration TWP groups did not exhibit significant effects on these enzymes. Additionally, star plot analysis revealed that the high-concentration TWP groups significantly affected the level of GSH and MDA in mussels (Figure 5A–C).

**Figure 6:**
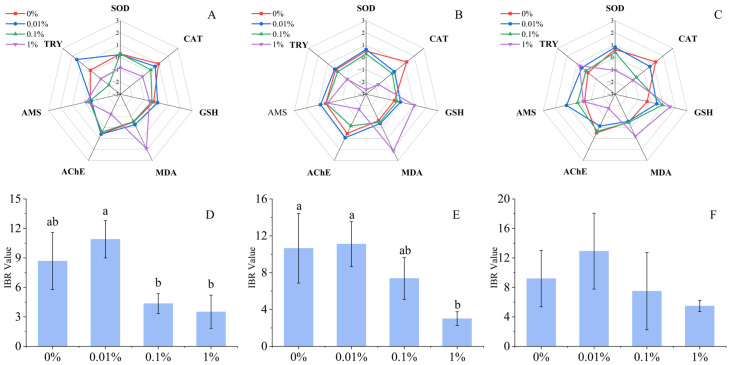
Star plots (**A**–**C**) and corresponding IBR values (**D**–**F**) of physiological and biochemical indices in mussels exposed to TWP leachates for 7, 14, and 21 days ((**A**,**D**): 7 days; (**B**,**E**): 14 days; (**C**,**F**): 21 days, different letters mean there are significant differences between control groups and experimental groups).

The exposure to TWP leachates significantly affected enzyme activities in mussels compared to the control group. At the high concentration, TWP leachates exerted significant effects on enzymes associated with physiological oxidative stress, digestion, and neurotoxicity on days 7 and 14 (Figure 6D,E). However, these effects were no longer significant on day 21 (Figure 6F). The star plots exhibited similar variation trends in GSH and MDA. The high-concentration TWP leachate group also induced notable changes in GSH and MDA levels (Figure 6A–C), which were consistent with the patterns observed in the TWP groups.

### 3.4. Effects of TWPs and Leachates on Gut Microbiota

#### 3.4.1. Effects of TWPs and Their Leachates on the Alpha Diversity

**Figure 7:**
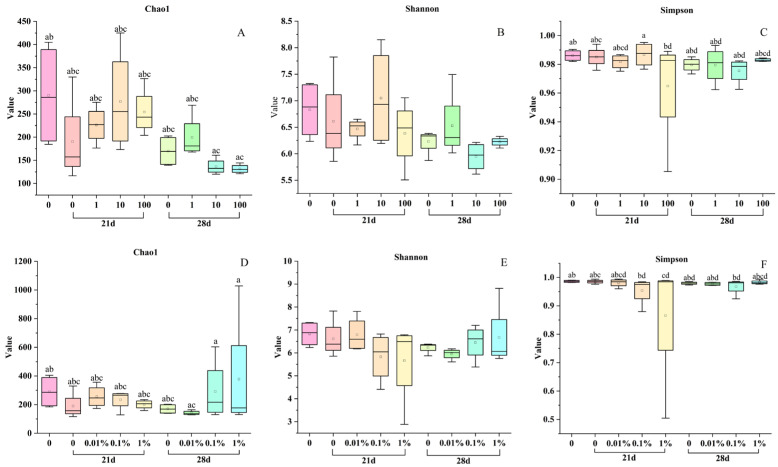
Alpha diversity index analysis of mussel gut microbiota exposed to tire particles and their leachates. (**A**–**C**) Tire particles: (**A**) Chao1; (**B**) Shannon; (**C**) Simpson; (**D**–**F**) Tire leachates: (**D**) Chao1; (**E**) Shannon; (**F**) Simpson (different letters indicate significant differences between the control and experimental groups).

The Alpha diversity index was employed to analyze the richness and diversity of the gut microbiota in mussels. Analysis of the Chao1 index (Figure 7A) revealed a decrease in the gut microbiota richness across all TWP and TWP leachate groups. Following the 7-day recovery period, the Chao1 index increased in the medium and high-concentration TWP leachate groups, though these increases were not statistically significant. Analysis of the Shannon index (Figure 7B) showed an increase in this index in the medium-concentration TWP group and the low-concentration TWP leachate group. After the 7-day recovery period, the results for the Shannon diversity index were consistent with those of the Chao1 index. For the Simpson diversity index (Figure 7C), the high-concentration TWP leachate group had the highest Simpson index value, which was significantly different from the control group (*p* < 0.05), whereas no significant differences were observed among other groups.

In the TWP leachate groups, the Chao1 index (Figure 7D) also indicated an overall decrease in gut microbiota richness. However, following the 7-day recovery period, the medium- and high-concentration TWP leachate groups exhibited an increase in the Chao1 index, though these increases were not statistically significant. The Shannon index (Figure 7E) was elevated in the low-concentration TWP leachate group. For the Simpson diversity index (Figure 7F), the high-concentration TWP leachate group had the highest index value, which was significantly different from that of the control group (*p* < 0.05), whereas no significant differences were detected among the other TWP leachate groups.

#### 3.4.2. Effects of TWPs and Their Leachates on the Intestinal Microbial Community Composition at the Phylum and Genus Level

**Figure 8:**
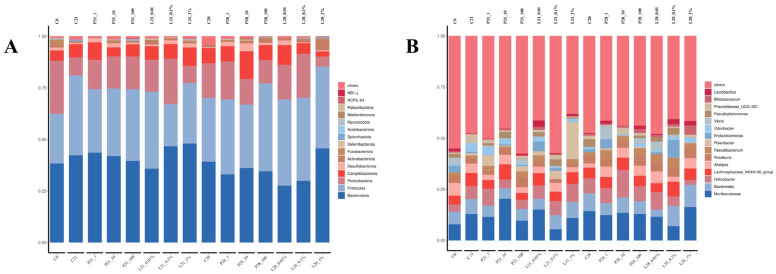
Relative abundance (**A**) Phyla level; (**B**) Genus level of mussel gut microbiota (C, P, and L, respectively represented the control group, TWP group, and leachate group).

The composition of the top 15 bacterial phyla in all samples is presented in Figure 8A. The dominant bacterial phyla in the mussel intestinal tract were Bacteroidota, Firmicutes, Proteobacteria, and Campylobacterota. Compared with the control group, the relative abundance of Bacteroidota generally decreased in the TWP groups, except in the medium-concentration group on day 21. The proportion of Firmicutes was reduced across all TWP groups. In contrast, the proportion of Proteobacteria increased in the TWP groups. Following the 7-day recovery period, the proportion of Firmicutes increased in most TWP groups, except in the low-concentration TWP group, while the changes in the proportion of Campylobacterota varied among different groups.

In the TWP leachate groups, the relative abundance of Bacteroidota generally increased, except in the low-concentration group on day 21. The proportion of Firmicutes was reduced across all TWP leachate groups. The proportion of Proteobacteria increased in most TWP leachate groups, except in the high-concentration group on day 21. Following the 7-day recovery period, the proportion of Bacteroidota decreased in most TWP leachate groups, except in the high-concentration group, while the proportion of Firmicutes increased in most TWP leachate groups, except in the medium-concentration group. The proportion of Campylobacterota decreased in the TWP leachate groups on day 21, except in the low and high-concentration groups, and increased in other TWP leachate groups.

The results for the top 15 most abundant genera in the intestinal microbial community are presented in Figure 8B. Muribaculaceae and Bacteroides were the two dominant genera at the genus level in the mussel gut. Compared with the control group, in the TWP groups, the relative abundance of Muribaculaceae increased in the medium-concentration group on day 21, but decreased in the low and high-concentration groups on day 21. The relative abundance of Bacteroides was reduced across all TWP groups. Following the 7-day recovery period, the relative abundance of Muribaculaceae increased in most TWP groups, except the high-concentration TWP groups.

Compared with the control group, in the TWP leachate groups, the relative abundance of Muribaculaceae decreased in the medium and high-concentration groups on day 21, but increased in the low-concentration group on day 21. The relative abundance of Bacteroides was reduced in the low and medium-concentration groups on day 21, whereas it increased in the high-concentration group on day 21. Following the 7-day recovery period, the relative abundance of Muribaculaceae increased in most TWP leachate groups, except the high-concentration group. In contrast, the relative abundance of Bacteroides decreased in the TWP leachate groups, with the exception of the medium and high-concentration groups.

#### 3.4.3. Functional Prediction of the Gut Microbiota in Mussels Exposed to TWPs and Their Leachate

**Figure 9:**
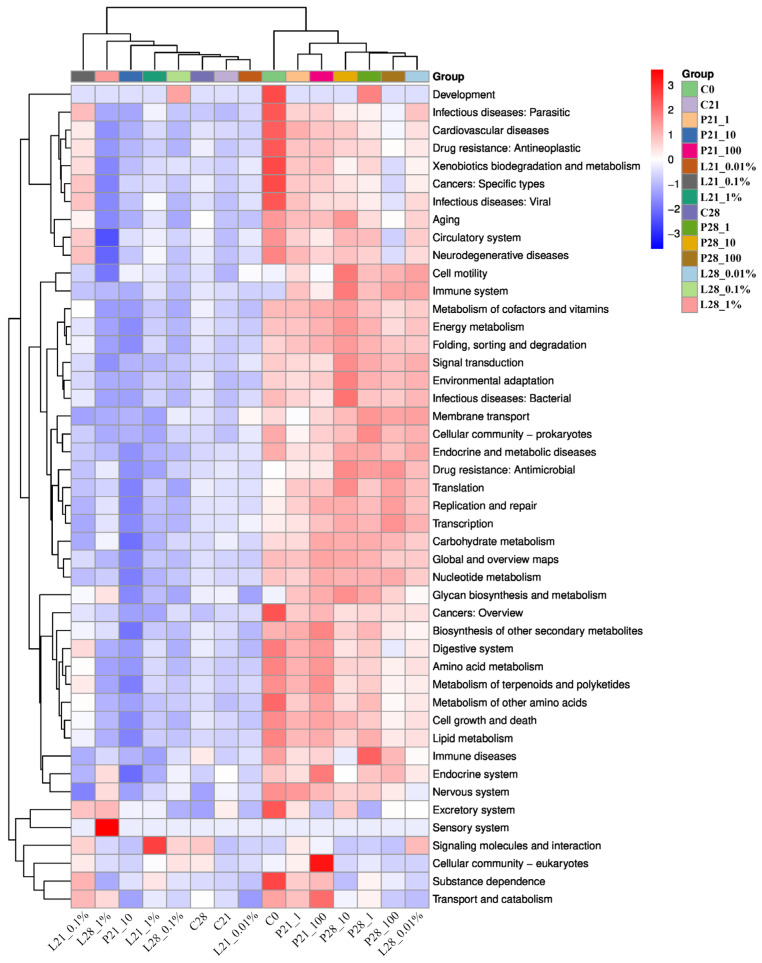
The clustering heat graph of the difference results from the KEGG function predicting mussel gut microbes (C, P, and L, respectively, represented the control group, TWP group, and leachate group).

Functional predictions of the intestinal microbial community based on KEGG Level 2 pathways are presented in Figure 9. After 21 days of TWP exposure, the predicted functional pathways in the medium-concentration TWP groups exhibited a downward trend compared to the control group, whereas those in the low and high-concentration TWP groups showed an upward trend. Following the 7-day recovery period, all predicted functional pathways across the TWP groups displayed an overall upward trend.

In the TWP leachate groups, metabolic pathways, including amino acid metabolism, energy metabolism, and lipid metabolism, were significantly upregulated in the medium-concentration group relative to the control group. After the 7-day recovery period, the membrane transport pathway was downregulated in the high-concentration TWP leachate groups, while the aforementioned metabolic pathways (amino acid, energy, and lipid metabolism) were upregulated in the low-concentration TWP leachate groups.

## 4. Discussion

### 4.1. Characteristics of TWPs and Their Leachates

TWPs were composed of a mixture of natural rubber (NR) and synthetic rubbers such as styrene-butadiene rubber (SBR), with the proportion varying by tire type and brand. For example, the SBR/(SBR + NR) ratio was 71.8% on average for passenger car tires but dropped to 20.7% for heavy-weight truck tires, which indicated that the proportion of SBR and NR significantly varied by vehicle and tire type [29]. These compositional differences dictated the material’s cross-linking density and elasticity, which fundamentally influenced how tires degrade and fragment under environmental stress, consequently leading to significant variations in the particle size distribution and surface morphology of TWPs [30]. Mechanical abrasion between these rubber compounds and road surfaces generated a mixture of micro- and nano-scale particles [1]. This size range overlapped with the preferred food size of many filter-feeding organisms [31]. Furthermore, previous studies have demonstrated that irregularly shaped microplastics with sharp edges and high surface roughness induced significantly greater physical abrasion and damage to the intestinal epithelium compared to smooth, spherical particles [32]. The irregular morphology of TWPs caused direct physical abrasion to the digestive tract upon ingestion [33].

Chemical analysis of the TWP leachates revealed a complex mixture predominantly consisting of heavy metal ions and organic pollutants. Among all the heavy metals, Zn^2+^ was the most abundant heavy metal, while PAHs, vulcanizing agents, and antioxidants like 6PPD constituted the primary organic fractions. Although these organic additives contribute to environmental contamination, Zn^2+^ has been identified as the primary driving factor for the chemical toxicity and environmental risk of the leachates [34]. This dominance was fundamentally rooted in the high loading of ZnO as a critical vulcanization activator during manufacturing, combined with the high aqueous solubility and superior bioavailability [35]. Notably, the concentration of Zn^2+^ detected in this study was relatively high compared to values reported in previous research. For instance, studies by Jeong et al. [29] and Shin et al. [23] reported Zn^2+^ concentrations in TWP leachates ranging from approximately 50 to 500 μg/L under similar experimental setups. This discrepancy could be attributed to a combination of experimental and material factors. TWPs used in this study were predominantly in the 0–30 μm range, providing a significantly higher specific surface area that facilitated more extensive leaching compared to larger fragments [36]. Furthermore, the extended leaching duration of 14 days employed in our methodology likely enhanced the extraction efficiency of Zn^2+^ [37].

### 4.2. Oxidative Stress Exposed to TWPs and Leachates

Oxidative stress was a critical toxic pathway in organisms, where the antioxidant defense system serves to counteract reactive oxygen species (ROS) and prevent cellular damage [14,38]. Our results revealed that the defense system was severely compromised following exposure to TWPs and their leachates. The primary enzymatic defense line collapsed, as evidenced by the significant inhibition of SOD and CAT activities. In TWP groups, the inhibition was likely attributed to the physical abrasion caused by the ingestion of TWPs. The rough and uneven surface of TWPs caused direct physical injury to gut tissues, leading to persistent mechanical inflammation and a massive burst of ROS. This excessive accumulation of ROS exceeded the compensatory capacity of the enzymatic defense, ultimately leading to the depletion and subsequent inhibition of these key antioxidant enzymes [39]. For leachate groups, the suppression of SOD and CAT was primarily driven by chemical components, notably high concentrations of Zn^2+^. Zn^2+^ disturbed the active sites of enzymes, inhibiting their catalytic activity [15]. Additionally, organic additives like PAHs in the leachates underwent metabolic activation, generating redox-active intermediates that contributed to oxidative stress [40].

In response to the escalating ROS levels, the mussels mounted a strong compensatory response, demonstrated by the significant upregulation of GSH. GSH served as a secondary defense by scavenging free radicals and maintaining intracellular redox homeostasis [41]. MDA was a terminal product of lipid peroxidation, serving as a reliable biomarker for oxidative damage to cell membranes [42]. In TWP groups, the upregulation of GSH indicated a strong compensatory cellular adaptive response to physical stress. It has been reported that mechanical damage induced by microplastic ingestion led to increased GSH levels in zebrafish as an adaptive response to mitigate associated oxidative injury [43]. Consistent with this mechanism, our research indicated that the mechanical damage likely triggered a substantial ROS burst, prompting GSH synthesis as a secondary defense line to mitigate oxidative injury. The significant increase in MDA levels in high-concentration TWP groups confirmed that the oxidative insult from physical damage was substantial enough to cause membrane lipid degradation. For leachate groups, the elevation of GSH was a response to chemical-induced oxidative stress. Zn^2+^ and organic compounds promoted ROS generation [44], leading to the upregulation of GSH synthesis as a defense mechanism. The significantly elevated MDA levels corroborate that the chemical insult induced significant lipid peroxidation [42].

### 4.3. Neurological Function, Digestive Functions, Exposed to TWPs and Leachates

AChE is a key enzyme responsible for terminating nerve signal transmission by degrading the neurotransmitter acetylcholine (ACh) [45]. The inhibition of AChE activity can result in the accumulation of neurotransmitter ACh in the nervous system, leading to overstimulation of muscarinic and nicotinic receptors, loss of nerve conduction in the organism, and motor dysregulation [46]. In this study, high-concentration TWP exposure significantly suppressed AChE activity. This neurotoxic effect likely originated from the systemic consequences of physical ingestion. The mechanical abrasion and persistent inflammation of the gut tissues induced by the rough TWPs triggered a systemic inflammatory response and a massive release of ROS. These oxidative stressors indirectly interfere with neural tissue integrity or inhibit enzyme activity through oxidative damage to the protein structure [47]. For example, polystyrene MPs have been shown to reduce AChE activity in juvenile guppies and zebrafish [43,48], potentially via induction of oxidative stress, inflammatory responses, or physical interference with neural tissue integrity. For leachate groups, Zn^2+^ has been demonstrated to directly inhibit AChE activity, possibly by interacting with the enzyme active site or by inducing conformational changes [49,50]. Furthermore, organic pollutants such as PAHs and benzothiazoles in the leachates also contributed to neurotoxicity through mechanisms involving oxidative stress induction or direct interaction with the neural system [51].

AMS and TRS activities are commonly used as indicators of digestive system status [52]. In TWP groups, no significant change in AMS activity was observed. This may be attributed to the relatively stable nature of amylase or its lesser sensitivity to particle-induced physical disruption, as AMS primarily catalyzes the hydrolysis of starch and may be less affected by direct particle interactions compared to proteolytic enzymes [53]. This may be attributed to the more hydrophilic nature of AMS substrates compared to the hydrophobic domains commonly targeted by proteases, rendering TWPs’ surfaces less interactive with starch-digesting enzyme systems [54]. In contrast, TRS activity was significantly reduced only in the high-concentration particle group, indicated impaired proteolytic function, possibly due to direct adsorption or inactivation of the enzyme by particle surfaces [55]. This impairment hindered normal protein digestion and further explained the observed growth abnormalities in the particle-exposed groups [56]. For leachate groups, the inhibition of TRS was attributed to Zn^2+^ and organic compounds, which interfered with the synthesis and secretion pathways within the digestive gland’s secretory cells, or disrupted the enzyme’s catalytic efficiency through metal-ion interference [57]. Similar to our conclusion, Xie et al. [58] found that exposure to heavy metals like Zn^2+^ has been shown to inhibit key digestive enzyme activities, thereby impairing nutrient absorption and energy metabolism.

The star plots and corresponding IBR values highlighted that TWPs exerted a more profound integrated impact on the antioxidant defense system (particularly GSH and MDA), digestive enzymes (TRS), and neurotoxicity biomarker (AChE) compared to their leachates. This enhanced toxicity of particles can be attributed to a physical mechanism. The physical presence of the particles likely caused abrasion and damage to gill and gut tissues upon ingestion, leading to direct tissue injury and inflammation [59].

### 4.4. Community Structure and Composition of Gut Microbiota

The Alpha diversity results showed that exposure to TWPs and their leachates reduced the richness of the gut microbiota in mussels, but the patterns of their effects on the community structure were different. The change in the Simpson index indicated that at medium concentrations of TWP exposure, the species evenness actually increased. This suggested that the physical presence of TWPs might inhibit some species through occupying ecological niches and causing physical damage, thereby allowing the relatively dominant strains with higher tolerance to rise [60]. This physical disturbance pattern did not show a significant increase in diversity during the recovery period, indicating that the physical impact of particles may cause relatively long-lasting structural changes to the intestinal microenvironment, hindering the rapid recovery of the community [61]. In contrast, after exposure to the TWP leaching solution, the general decline in the Chao1 index indicated that the selective pressure of the chemical substances was more significant, inhibiting the sensitive bacterial strains and resulting in a significant reduction in diversity. However, after the 7-day recovery period, the Chao1 index showed a recovery trend. This suggested that the toxic effect of the leachates was mainly driven by bioavailable chemicals (such as Zn^2+^, PAHs), and its influence was to some extent reversible. Once the chemical stressor was removed, the microbial community had the potential to regain its original richness.

At the phylum level, exposure to TWPs led to a general decrease in the relative abundance of the Bacteroidota phylum and an increase in the Proteobacteria phylum. The abnormal proliferation of the Proteobacteria phylum was usually associated with intestinal inflammation and ecological imbalance [62]. For example, Shi et al. [63] also observed that exposure to microplastics increased Proteobacteria in zebrafish. This microbiota imbalance was potentially related to oxidative stress and impaired digestive function. The increase in Proteobacteria may exacerbate oxidative damage to the intestinal epithelium, and the inhibition of key digestive enzyme activity may be related to the reduction in the production of intestinal microbial metabolites or changes in the microbial community structure that lead to changes in the digestive enzyme secretion environment. In the TWP leachates, the abundance of the Firmicutes phylum was consistently decreased in all groups. The Firmicutes phylum is a key beneficial phylum in bivalve animals that is closely related to immune function and maintenance of intestinal homeostasis [47]. Our results suggested that the significant reduction in Firmicutes may be linked to Zn^2+^. Zn^2+^ has been demonstrated to exhibit bacteriostatic effects and selectively inhibit certain beneficial bacterial communities [64].

At the genus level, exposure to TWPs led to a significant reduction in the relative abundance of beneficial bacteria such as Bacteroides across all exposure groups. Bacteroides plays a crucial role in carbohydrate metabolism and nutrient processing for the host [65]. Its depletion suggested a disruption in the digestive assistance, potentially exacerbating the observed digestive dysfunction. Concurrently, an increase in the relative abundance of other genera was observed, such as Muribaculaceae in the medium-concentration group. The proliferation of these genera was often associated with intestinal inflammation and ecological dysbiosis, and they may be more adapted to thrive in the compromised intestinal environment caused by physical particle abrasion and oxidative stress [66]. In the TWP leachate groups, a significant increase in potential pathogenic bacteria like Pseudomonas was noted, further intensifying the physiological burden on the host. The proliferation of pathogenic bacteria triggered an immune response that consumed a large amount of energy [43], which is consistent with the upregulation of multiple metabolic pathways observed. Zhang et al. [43] found that in zebrafish exposed to TWPs, the increase in potential pathogenic bacteria was accompanied by changes in the expression of genes related to energy metabolism. Both the host and the microbial community may need to mobilize additional metabolic resources to cope with the infection pressure and chemical toxicity, leading to an imbalance in energy distribution. In summary, TWPs primarily induced physical damage that altered the microbial landscape, favoring genera adapted to a stressed niche, while TWP leachates exerted chemical stress that can selectively promote an increase in specific potential pathogens like Pseudomonas.

### 4.5. Functional Changes in Gut Microbiota

Functional prediction of the gut microbiota based on KEGG Level 2 pathways revealed significant changes in response to exposure to TWPs and leachates. In TWP groups, the most prominent upregulation was observed in membrane transport pathways, reflecting an active microbial defense against the physical presence of particles. This likely involved the export of abrasion-induced cellular debris and the attempted import of nutrients in a gut environment [67]. Meanwhile, there was a widespread upregulation of core metabolism pathways. The increase in lipid metabolism was a direct response to the oxidative stress, facilitating the detoxification of lipid peroxidation products and membrane repair necessitated by physical damage. The upregulation of energy metabolism, amino acid metabolism, and carbohydrate metabolism indicated the microbiota was under high energy demand to repair damage, and potentially compensate for the impaired digestive function caused by particle interference [68]. Enhanced signal transduction suggested the microbial community was intensifying cell–cell communication to coordinate a collective survival strategy in this physically altered niche [69]. Similar to the metabolic disturbances observed in zebrafish exposed to polyethylene microplastics, where pathways related to lipid and fatty acid metabolism were significantly disrupted [70], the upregulation of these metabolic pathways highlighted a microbial response to particle-induced stress, aimed at maintaining energy homeostasis amidst gut dysbiosis. Exposure to TWP leachates also altered microbial function, but the pattern was more indicative of a chemical stress response. The upregulation of membrane transport pathways, particularly those for ion transport and efflux pumps, was directly countering the chemical toxicity of bioavailable ions like Zn^2+^ and organic pollutants by enhancing their export from microbial cells [71]. The upregulation in metabolism pathways, such as lipid and amino acid metabolism, was also linked to oxidative stress and detoxification of specific chemical compounds.

## 5. Conclusions

The findings of this study demonstrated that TWPs posed a significant threat to the health of *Mytilus coruscus*. The observed toxicity, characterized by growth inhibition, oxidative stress, neurotoxicity, and digestive dysfunction, was more pronounced upon exposure to TWPs compared to their leachates. This finding underscored that the toxicity of TWPs cannot be attributed solely to the chemical components released; the physical presence of the particles played a critical role. Furthermore, the disruption of the gut microbiota suggested a broader sublethal impact on host physiology and homeostasis. Therefore, reducing the release of TWPs into aquatic environments is crucial for the conservation of marine organism health.

## 6. Limitations

The exposure duration (21 days) represents a short-to-medium-term chronic exposure; longer-term studies are required to assess multi-generational effects or permanent ecological shifts.

## Data Availability

The original contributions presented in this study are included in the article. Further inquiries can be directed to the corresponding author.

## Supplementary Materials

The following supporting information can be downloaded at: https://www.mdpi.com/article/10.3390/toxics14060468/s1, Text S1: Characterization of TWPs leachate; Figure S1: Size of Tire Wear Particles (TWPs); Figure S2: Influence to the growth rate of mussels in different groups of (A) TWPs; (B) leachates to mussels; Table S1: The content of polycyclic aromatic hydrocarbons (PAHs) and heavy metal pollutants in the stock solution of TWP leachates; Table S2: P values for the statistical analysis of oxidative stress and enzyme activity biomarkers in mussels exposed to TWPs and their leachates.
