# Supplementary material for: The Toxicity of Tire Wear Particles and Their Leachates on Digestion and Gut Microbiota of Mytilus coruscus
Source: Toxics. 2026 May 27;14(6):468. doi: 10.3390/toxics14060468 (PMC13307113; doi:10.3390/toxics14060468)
Supplement: Supplementary file 1 [file toxics-14-00468-s001.zip › Supplementary Materials.pdf]

Text S1. Characterization of TWPs leachate

Prior to instrumental analysis, organic compounds were extracted from the leachate using a solvent-based method. Specifically, 500 μL of an extraction solvent mixture (dichloromethane:n-hexane = 1:1) and an internal standard (naphthalene-d8, final concentration 2 μg/mL) were added to the medium, both before and after exposure. The mixture was vortexed for 1 minute, followed by phase separation via centrifugation (2000 rpm, 2 minutes). This extraction was performed in triplicate, and the combined organic phases were dried over anhydrous Na_2SO_4 before analysis.

The extracts were analyzed by GC-MS (7890B/5977A with EI ion source, Agilent Technologies Inc., USA) equipped with a DB5-MS column, operating in full scan mode. The oven temperature was held at 60°C for 2 minutes, then ramped to 300°C at 6°C/min and held for 12 minutes. Mass spectra were acquired after a 6-minute solvent delay in full scan mode (m/z 50-500) and processed using MassHunter Workstation Software B.08.00. Detected compounds were identified by matching against the NIST 14.0L library via the MassHunter Unknowns Analysis software. Based on the full-scan results, standard solutions of target additives at five concentration levels were analyzed in Selected Ion Monitoring (SIM) mode to establish linear calibration curves. The recovery rates of the extraction method for these additives were validated. Additive concentrations in the samples were subsequently quantified using these calibration curves.

For metal analysis, the leachate or medium was diluted to a final volume of 10 mL with 10% nitric acid (to ensure complete metal dissolution) and spiked with ^{103}Rh as an internal standard. Metal ion concentrations were determined using ICP-MS (iCAP™ TQe, Thermo Fisher Scientific, USA) and calculated based on the internal standard peak area.

Table S1 The content of polycyclic aromatic hydrocarbons (PAHs) and heavy metal pollutants in the stock solution of TWP leachates.

Name	Concentration (μg/L)
Naphthalene	38.89
2-Methyl naphthalene	31.00
Phenanthrene	19.04
Pyrene	14.52
Fluorene	8.94
Benzo(g,h,i)perylene	6.75
Acenaphthene	3.97
Benz(a)anthracene	2.25
Fluoranthene	1.17
Zn^{2+}	1289.99
Cu^{2+}	7.92

Cr^{2+}	1.07
Ni^{2+}	0.81

Table S2 P values for the statistical analysis of oxidative stress and enzyme activity biomarkers in mussels exposed to TWPs and their leachates

	form	D7	D14	D21
SOD	particle	< 0.001	0.001	< 0.001
	leachate	0.004	< 0.001	< 0.001
CAT	particle	< 0.001	< 0.001	< 0.001
	leachate	0.002	0.002	0.007
GSH	particle	< 0.001	< 0.001	0.002
	leachate	0.467	0.007	0.248
MDA	particle	< 0.001	< 0.001	0.003
	leachate	< 0.001	< 0.001	0.066
AMS	particle	0.045	0.764	0.548
	leachate	0.960	0.539	0.442
TRY	particle	< 0.001	< 0.001	0.002
	leachate	0.003	0.718	0.494
AChE	particle	0.003	< 0.001	< 0.001
	leachate	< 0.001	0.005	0.004

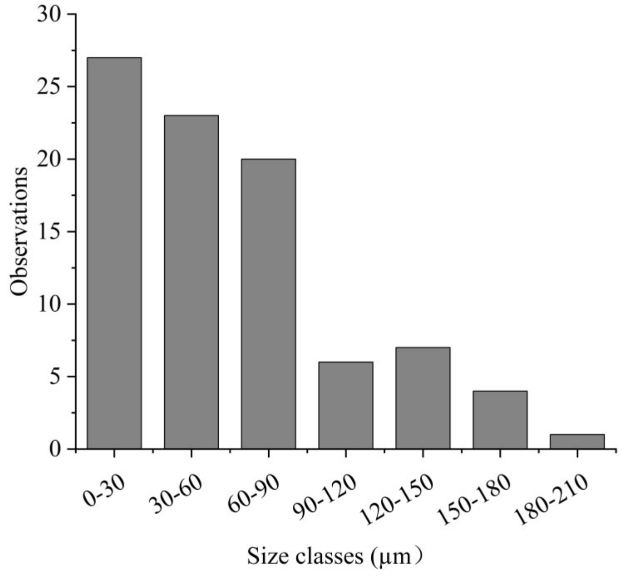

Figure S1 Size of Tire Wear Particles (TWPs)

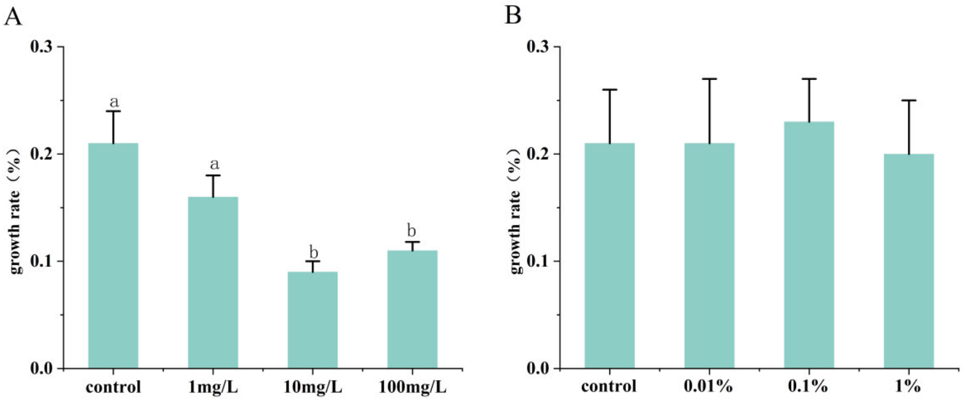

Figure S2 Influence to the growth rate of mussels in different groups of (A) TWPs; (B) leachates to mussels.
